# Stability and Change in Wellbeing Throughout Adolescence and Its Relationship with Life Events: A Longitudinal Twin Study

**DOI:** 10.1007/s10519-026-10262-4

**Published:** 2026-04-11

**Authors:** Eirunn Skaug, Trine Waaktaar, Svenn Torgersen

**Affiliations:** Department of Psychology, University of Oslo, Forskningsveien 3A, 0373 Oslo, Norway

**Keywords:** Adolescent wellbeing, Genetic influences, Environmental factors, Longitudinal twin study, Life events

## Abstract

Wellbeing—the balance of positive and negative affect and life satisfaction—is closely tied to mental health. However, the sources of stability and change and how life events shape wellbeing are not well understood. Data from the *Oslo University Adolescent and Young Adult Project* (2,879 individuals from 1,483 families) provided wellbeing and life‑event data across three waves ~ 2 years apart (ages 12–18 at Wave 1). We used a trivariate Cholesky decomposition to estimate genetic and environmental contributions to wellbeing, and genetically informative random intercept cross‑lagged panel models (RI‑CLPMs) to test prospective effects of life events on wellbeing. Wellbeing showed moderate stability, predominantly driven by genetic influences (81%). Cross-sectional heritability was about 50% at Waves 1–2 and declined to 26% at Wave 3. Negative life events were correlated with lower wellbeing (concurrent *r* = − 0.34 to − 0.28; 2-year *r* = − 0.26 to − 0.19; 4-year *r* = − 0.22 to − 0.12). However, RI‑CLPMs indicated minimal longitudinal effects of life events on wellbeing after accounting for genetics and trait stability: negative dependent life events explained 1% of change from Wave 1 to Wave 2 and ≈ 0% (non-significant) from Wave 2 to Wave 3. Genetic factors primarily account for stability in wellbeing, while measured life events showed limited prospective influence on wellbeing levels. Future research should combine richer genetically informed longitudinal designs (e.g., measured genotypes and GxE models) and examine biological pathways and person-level processes (e.g., neural and epigenetic mechanisms) to clarify pathways influencing wellbeing and inform interventions.

**Supplementary Information:**

The online version contains supplementary material available at 10.1007/s10519-026-10262-4.

## Introduction

Wellbeing can be defined as an individual’s evaluation of their overall life satisfaction, the presence of positive emotions, and the relative absence of negative emotions (Diener et al. 2018). Terms such as subjective wellbeing, wellbeing, life satisfaction, quality of life, and happiness are often used interchangeably, as they all focus on aspects related to good mental and physical health. Despite nuanced differences in definitions and operationalizations across various contexts and academic fields, these concepts fundamentally reflect the perception of a fulfilling and balanced life. Higher wellbeing is associated with favorable life outcomes, including better mental and physical health, increased longevity and reduced mortality (Bartels et al. 2013; Chida and Steptoe 2008; Sadler et al. 2011; Steptoe et al. 2015). Conceptualizing wellbeing as a substantive dimension of mental health extends the construct beyond the mere absence of distress symptoms. Wellbeing is found to be moderately stable over long periods of time, with test-retest correlations of approximately 0.40 to 0.60 over 10-years intervals, and around 0.30 for intervals of 20 years or more (Anusic and Schimmack 2016; Lucas and Donnellan 2007; Schimmack et al. 2010). While most research on wellbeing focuses on adulthood and uses cross-sectional methods, work on adolescent wellbeing typically emphasizes developmental change. Prospective studies that have included adolescence specifically suggest a reduced wellbeing across the developmental phase compared to any other phase (Buecker et al. 2023; Orben et al. 2022), specifically with indications of stage-related drop in stability in the transition between late childhood and young adolescence (Casas and González-Carrasco 2019) as well as from late adolescence into young adulthood (Chen and Page 2016). Such reductions seem to be related to increased adolescent life stress (Diener and Diener McGavran 2008; Ouyang et al. 2021; Petersen et al. 2022; Willroth et al. 2021). However, although the genetic and environmental architecture of these associations are highly relevant for prevention and treatment intervention purposes, the nature of their association is largely unknown. In cross-sectional data, the heritability of wellbeing is typically estimated to be around 40% (Baselmans et al. 2018; Nes and Røysamb 2015), varying somewhat between countries, with a world-wide wellbeing estimated to be somewhat lower, between 31 and 32% (Bartels 2015; Røysamb et al. 2023). To examine genetic and environmental factors influencing wellbeing over time, longitudinal studies are needed. Longitudinal genetically informative studies of both adult and adolescent samples suggest that the cross-time stability of wellbeing is around 0.50, the stability primarily due to consistent genetic influences across time (de Vries et al. 2024; Lykken and Tellegen 1996; Nes et al. 2006).

As proposed within the general diathesis-stress framework for mental health (Zubin and Spring 1977), life events may move individuals above or below their baselines. Stressful experiences such as death or illness of a close one, worsening in financial situation, and injury/illnesses have been shown to affect mental health and wellbeing in longitudinal studies of adult samples (Kettlewell et al. 2020; Luhmann et al. 2012). Adolescence is a phase with significant stress related to developmental change, both physical, mental, interpersonal and social (Mastorci et al. 2024; Sisk and Gee 2022). It is also associated with marked drop in wellbeing, which is a general global trend (Handa et al. 2023). People often believe that specific life events significantly affect our wellbeing. For instance, it might be assumed that a teenager joining an enjoyable group of friends would become happier, while negative occurrences, such as arguments with siblings or peers, would harm their wellbeing. Although widely regarded as clinically important, life events do not uniformly produce the expected outcomes: on average their effects on wellbeing are modest and often short-lived, with substantial individual variability (Cohen et al. 2019; Suh et al. 1996). Studies considering genetic factors indicate that many life events are not merely passive occurrences but are influenced by individual behavior and personality traits, which are genetically influenced (Kendler and Baker 2007; Plomin et al. 1977; Vinkhuyzen et al. 2010). Further indications of the close entanglement between life events and people’s traits, studies have found that life satisfaction is prospectively predictive of life events (Luhmann et al. 2013), and perceptions of the impact of life stressors are more closely related to genetic factors influencing wellbeing than to the objective frequency of such events (Burns and Machin 2013).

A recent twin study investigating wellbeing across the lifespan found that genetic effects accounted for most of the stability in adolescence (de Vries et al. 2024). Nonetheless, there was also evidence of genetic innovation and gene-environment interaction effects that contributed to change. In contrast, the effect of the shared environment predominated in childhood, while nonshared environmental effects dominated intraindividual change in adulthood.

Life events do not necessarily have a unidirectional effect on us. Instead, the occurrence of an event itself may be related to our behaviors shaped by our genetic makeup (McAdams et al. 2013). Consequently, when studying the impact of life events on various outcomes, it is important to use designs that can control for the confounding effects of shared genetics. Twin studies have shown that wellbeing and life events share genetic influences (Wootton et al. 2017), most probably thorough mechanisms of gene-environment correlations where a positive life attitude trait leads people to seek and evoke positive experiences (Mann et al. 2019), and vice versa.

This study aims to enhance our understanding of wellbeing during adolescence and young adulthood, a critical period marked by significant changes in autonomy, social environments, and the onset of mental health disorders (Kessler et al. 2007). Our primary research questions focused on the contributions of genetic and environmental factors to stability and change in wellbeing within individuals across the sampled measurement intervals (i.e., three measurement waves, each approximately two years apart). Additionally, we investigated the relationships between wellbeing and life events to determine how significant experiences influence wellbeing. Identifying these dynamics is crucial for predicting individual differences in wellbeing and informing targeted prevention and intervention strategies to foster healthier developmental trajectories.

## Methods

### Sample and Procedure

The data come from the Oslo University Adolescent and Young Adult Twin Project (Torgersen and Waaktaar 2019, 2020). In this Norwegian population-based longitudinal twin study, all twins born in Norway from the birth cohorts 1988–1994 (seven cohorts) were invited to participate. The study was approved by the Norwegian Data Inspectorate and the Regional Committees for Medical and Health Research Ethics. American Psychological Association ethical principles were followed in the conduct of the study.

The present study utilized data from self-report questionnaires that were sent out to the twins three times throughout adolescence and young adulthood, starting when the twins were 12 to 18 years old, with two-year intervals between measurement waves. The mean age at the first, second and third wave were 15.2 years (*SD* = 1.9), 16.9 years (*SD* = 2.0), and 19.6 years (*SD* = 1.9), respectively. All twins who participated on at least one measurement wave were included in the analyses, resulting in an overall sample size of 2,879 twins (56% females) from 1,483 families, including 1,094 monozygotic (MZ) and 1,785 dizygotic (DZ) twins. Of the 2,879 participants, 1,132 twins took part in all three waves, 870 twins participated in two waves (613 in Wave-1 and Wave-2; 179 in Wave-1 and Wave-3; 78 in Wave-2 and Wave-3), and 877 twins participated in only one wave (710 in Wave-1; 97 in Wave-2; 70 in Wave-3). Number of complete and incomplete twin pairs at each wave are presented in Online Resource Table S1.

Zygosity was determined by a combination of questionnaire items and gene testing, and the misclassification rate has been estimated to be 0.45% (Skaug et al. 2022a, b). (L Currie evin and)

### Measures

Wellbeing was assessed using the Cantril ladder (Cantril 1965), one of the most widely used measures of wellbeing (Nilsson et al. 2024). Cantril ladder has shown good reliability and validity in adolescent samples (Levin and Currie 2014). The participants were asked to evaluate their lives by imagining a ten-step ladder representing their *worst* (1) to their *best* (10) possible life.

To assess a range of positive and negative life events in adolescence, the participants were asked if they had experienced any of 38 events over the past year (0 = *no*; 1 = *yes*). Twenty-nine of these events were derived from the Life Event Questionnaire for Adolescents (LEQ-A; Masten et al. 1994), and an additional nine events were included after a pilot study (the full list of events are presented in Online Resource Table S2). The life events were categorized into three groups: negative dependent, negative independent, and positive dependent events. Dependent events are those considered to be influenced by an individual’s behavior, whereas independent events are those believed to occur independently of an individual’s actions. Positive independent events were not represented in the set of life events, likely due to their rarity or because positive life events are generally perceived to be behavior-dependent (Kandler et al. 2012). The LEQ-A events were categorized according to Masten et al. (1994), while the additional events were classified based on the authors’ evaluation. For instance, “I was disappointed by a friend” was categorized as a negative dependent event, “one of my parents died” as a negative independent event, and “I got a new friend” as a positive dependent event. In total, 14 events were classified as negative dependent, 19 as negative independent, and five as positive dependent. For each participant, the total number of events reported within each category was calculated.

### Statistical Analyses

Pearson correlations between study variables were calculated to investigate phenotypic associations between life events and wellbeing, both cross-sectionally and over time. They were also used to assess the stability in wellbeing and life event cluster scores across time.

Biometric analyses, using structural equation models, were fitted to examine the nature of observed phenotypic associations. Models were applied to raw data using full information maximum likelihood with the R package OpenMx (Neale et al. 2016). Code for analyses is available at https://osf.io/w8tej/overview (Skaug, 2026). The classical twin design compares phenotypic similarity between monozygotic (MZ) and dizygotic (DZ) twins to estimate additive genetic (A), shared environmental (C), and nonshared environmental (E) contributions to variance and covariance. MZ twins are genetically identical; DZ twins share ~ 50% of segregating genes. Greater MZ than DZ similarity indicates A; DZ similarity greater than half the MZ similarity indicates C; differences within MZ pairs (including measurement error) indicate E.

As an initial step, we fitted a trivariate Cholesky decomposition model to decompose the variance in wellbeing at each wave into A, C and E components. A nested AE model was compared to the full ACE model using Akaike’s information criterion to select the best-fitting model (Akaike 1987).

### Cross-Lagged Panel Models

**Fig. 1:**
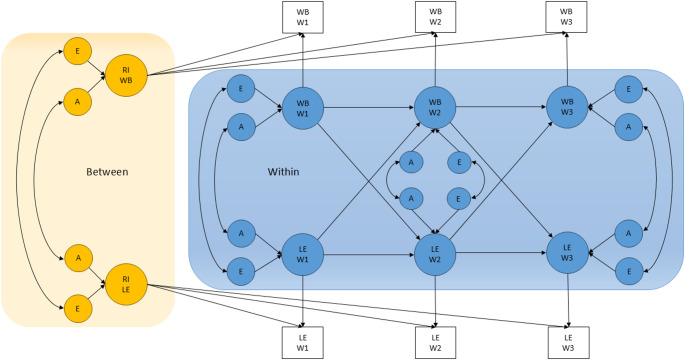
Genetically informative random intercept cross-lagged panel model. *WB* wellbeing, *LE* life events, *RI* random intercept, *A* additive genetic factors, *E* unique environmental factors, *W1* wave 1, *W2* wave 2, *W3* wave 3. Rectangles represent observed variables and circles represent latent variables. “Between” refers to between-person processes (time-invariant stability). “Within” refers to within-person processes (variance due to changes within individuals over time), including autoregressive paths (temporal stability) and cross-lagged paths (change).

Cross-lagged panel models examine longitudinal associations between variables to infer influences over time. The Random Intercept Cross-Lagged Panel Model (RI-CLPM), developed by Hamaker et al. (2015), separates variance into stable (between-person) and dynamic (within-person) components. Between-person variance is captured by random intercepts, reflecting stable influences (e.g., consistent wellbeing). Following Hamaker et al., factor loadings from the random intercepts to observed variables were fixed at one to denote time-invariant effects. Within-person latent factors for wellbeing and life events were modeled by specifying a latent variable for each observed measure, with all loadings set to one. These time-specific latent factors represent deviations from an individual’s stable mean. Autoregressive paths show the amount of carry-over of within-person deviations across waves (positive autoregressive paths indicate persisting deviations such as sustained elevated wellbeing). Cross-lagged paths estimate how within-person deviations in one variable predict subsequent changes in another, controlling for stability. Incorporating the genetically informative aspect into the RI-CLPM, we partitioned both the between-person (random intercepts) and within-person (wave-specific) variances into genetic and environmental components, allowing these components to contribute to stability, autoregressive, cross-lagged, and wave-specific variance. Thus, within-person variance at later waves reflects a combination of prior-wave influences transmitted via autoregressive and cross-lagged paths, together with new wave-specific genetic and environmental contributions. A description of these calculations are presented in Online Resource Figure S1. The full technical specification of the genetically informative RI-CLPM is provided in (Skaug et al. 2024) For simplicity, Fig. 1 illustrates the model for one twin only, and only A and E influences are included.

Absolute model fit was assessed using the comparative fit index (CFI), Tucker-Lewis index (TLI), and root mean square error of approximation (RMSEA), with CFI and TLI values above 0.95 and RMSEA values below 0.06 signifying good model fit (Hu and Bentler 1999). The significance of the autoregressive and cross-lagged parameters was tested by constraining them to zero in nested models and comparing fit to the full RI-CLPM using likelihood-ratio chi-square tests; a non-significant chi-square difference indicates no substantial loss of fit.

## Results

**Fig. 2:**
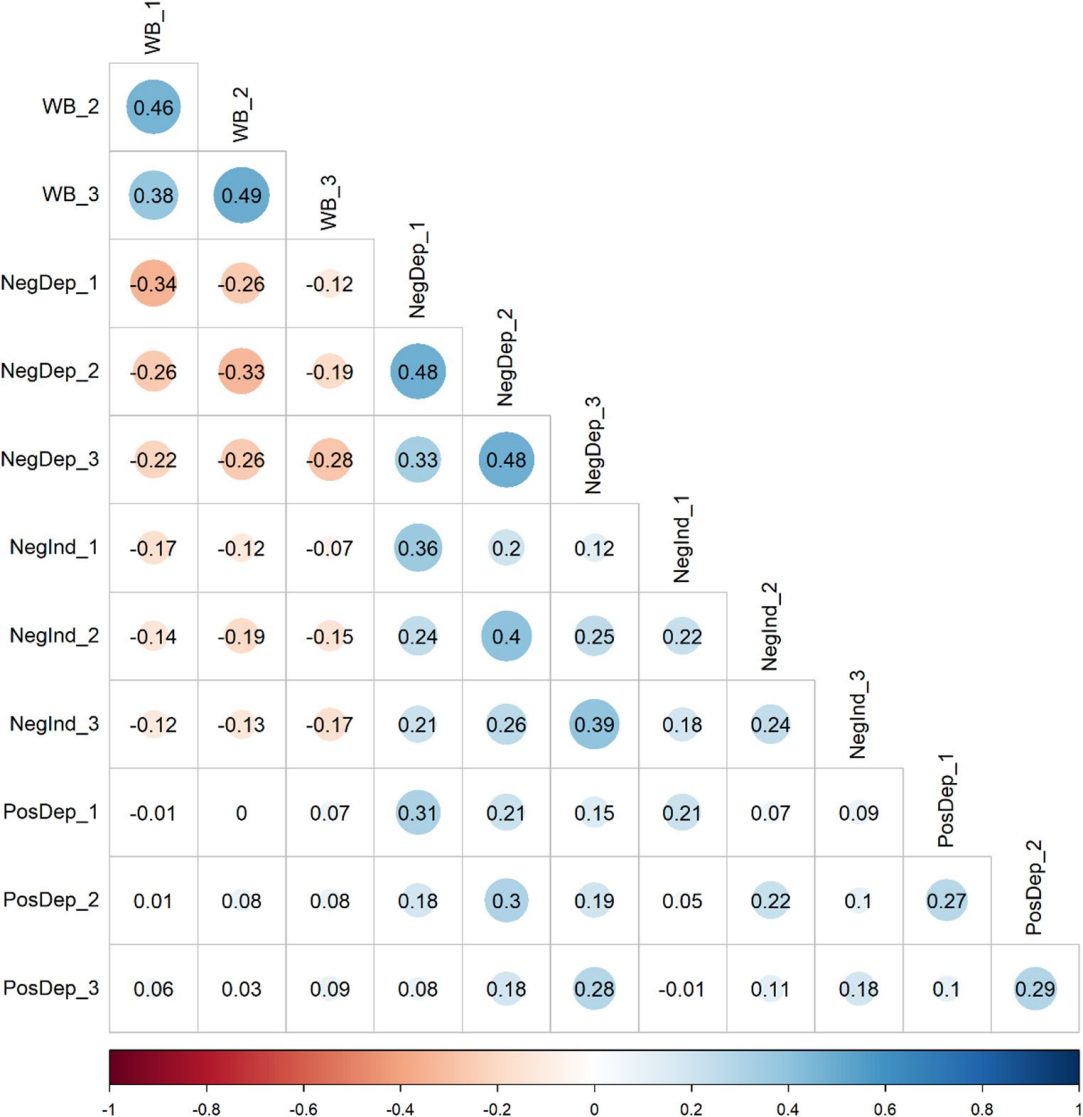
Phenotypic correlations between study variables. *WB* wellbeing, *NegDep* negative dependent life events, *NegInd* negative independent life events, *PosDep* positive dependent life events. Numbers indicate the different measurement waves (1–3). Cells without color indicate non-significant correlations (*p* > 0.05)

Correlations between all observed measures are presented in Fig. 2 (for descriptive statistics for the study variables, see Online Resource Table S3).

Wellbeing and negative dependent life events showed moderate within-trait correlations across measurement waves, indicating that both wellbeing and the number of reported negative dependent life events were relatively stable over time. Negative independent and positive dependent life events exhibited lower stability, as indicated by their lower within-trait correlations. Looking at the associations between wellbeing and the various life events clusters, both negative dependent and negative independent life events were negatively associated with wellbeing. In contrast, the correlations between positive dependent life events and wellbeing were negligible, indicating that the positive life events measured in this study were not related to level of wellbeing. Consequently, positive dependent life events were excluded from subsequent twin analyses examining the nature of the longitudinal relationship between life events and wellbeing.

### Biometric Models

First, a trivariate Cholesky decomposition model was fitted to determine the proportion of genetic and environmental variance in wellbeing at each measurement wave. Dropping the shared environmental parameters (C) resulted in a better fitting model compared to the full ACE model, as indicated by lowest Akaike’s information criterion value. Twin correlations and results from the AE model are presented in Table 1. Additive genetic influences accounted for a substantial proportion of individual differences in wellbeing at each time point. However, the heritability of wellbeing was markedly lower at the third measurement wave compared to the first two waves.

To examine whether within-wave age variation influence the heritability estimates, we performed additional analyses in which each wellbeing measure was regressed on participants’ age within the corresponding wave, and the residuals from these regressions were saved. These residuals (i.e., wellbeing scores with the effect of within-wave age removed) were then used as the observed variables in the same trivariate twin ACE/AE model described above. Results from these models showed close to identical heritability estimates to those reported for the raw scores (see Online Resource Table S4), suggesting that within-age variation does not influence the heritability estimates. This pattern could reflect an age-related decrease in the heritability of wellbeing between adolescence and young adulthood, but alternative explanations — such as cohort or study-wave differences — cannot be ruled out given the overlapping age ranges across waves.

**Table 1:** Twin correlations, heritability (a^2^) and proportion of unique environmental variance (e^2^) in wellbeing

Variable	Twin correlations		Model estimates	
	rMZ	rDZ	a^2^	e^2^
Wellbeing_1	0.50 [0.43, 0.56]	0.21 [0.14, 0.27]	0.48 [0.42, 0.54]	0.52 [0.46, 0.58]
Wellbeing_2	0.49 [0.40, 0.56]	0.22 [0.14, 0.30]	0.46 [0.39, 0.52]	0.54 [0.48, 0.61]
Wellbeing_3	0.28 [0.16, 0.39]	0.11 [0.01, 0.22]	0.26 [0.16, 0.36]	0.74 [0.64, 0.84]

95% confidence intervals in brackets. *rMZ* correlation within monozygotic twin pairs, *rDZ* correlation within dizygotic twin pairs. Wellbeing numbers indicate the different measurement waves (1–3)

Variance decompositions of the measured life events have been conducted in a previous study based on the same data set (Skaug et al. 2022a, b). Pertinent for the aims of the present study, the findings revealed that the number of negative dependent life events were moderately heritable across the measurement waves, with heritability estimates ranging from 47% to 55%. The remaining variance was attributed to unique environmental influences. In contrast, for the number of negative independent life events, shared environmental influences also contributed to the variance. Specifically, shared environmental influences accounted for between 29% and 49% of the variance, while heritability estimates ranged from 12% to 25%. Consequently, in the cross-lagged models, we estimated all three sources of variance (A, C and E influences) in the variance decomposition of negative independent life events, whereas only additive genetic and unique environmental influences were modeled in the variance decomposition of negative dependent life events.

### Genetically Informative Random-Intercept Cross-Lagged Panel Models

Genetically informative RI-CLPMs were fitted to data to examine stability and change in wellbeing, and to examine whether reported life events predicted change in levels of wellbeing. Separate models were fitted to negative dependent and negative independent life events. The RI-CLPMs showed good absolute fit (see Online Resource Table S5).

#### Stability and Change in Wellbeing

Table 2 presents the proportion of variance in wellbeing accounted for by the random intercept, which reflects time-invariant stability, along with the proportion of genetic and environmental influences contributing to this stability. The equations for calculating these proportions are given in Online Resource Figure S1. Wellbeing demonstrated a moderate level of stability, with the random intercept accounting for approximately 40% of the total variance at each measurement wave. The genetic and environmental variance in the random intercept reflects stable genetic and environmental factors exerting a continuous influence on wellbeing across all measurement waves. Genetic influences accounted for most of this time-invariant variance, explaining 81% of the variance in the random intercept.

**Table 2:** Time-invariant stability in wellbeing

Measure	Proportion of variance explained by the random intercept			Proportion of variance in the random intercept due to genetic and environmental influences	
	Wave 1	Wave 2	Wave 3	A	E
Wellbeing	40%	40%	36%	81%	19%

*A* additive genetic influences, *E* non-shared environmental influences

#### Change in Wellbeing

Although levels of wellbeing were relatively stable across measurement waves, a substantial portion of the total variance were attributed time-variant fluctuations (i.e., among 60% of the total variance at each measurement wave) (Table 2).

#### Relationship Between Life Events and Wellbeing

**Fig. 3:**
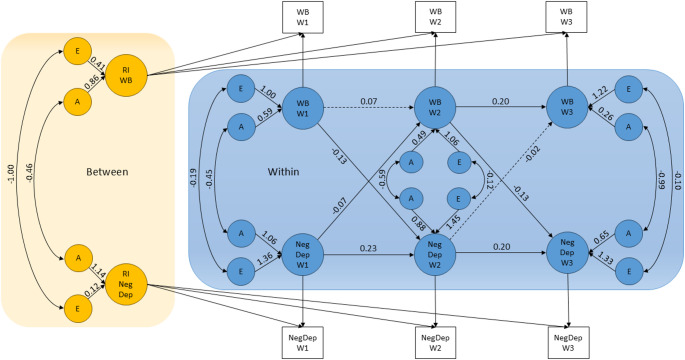
Path Diagram of the genetically informative RI-CLPM with unstandardized coefficients for the longitudinal relationship between wellbeing and negative dependent life events. Dashed lines indicate nonsignificant paths. A detailed description of model parameters is provided in Fig. 1. RI-*CLPM* random intercept cross-lagged panel model, *WB* wellbeing, *NegDep* negative dependent life events

The results of the cross-lagged analyses for dependent life events are shown in Fig. 3 (for results from the cross-lagged model for independent life events, see Online Resource Figure S2). All parameter estimates, with standard errors, are presented in Online Resource Table S6.

Standardized cross-lagged estimates for the relationship between negative life events and wellbeing are presented in Table 3 to enable comparison. All cross-lagged estimates were negligible in magnitude, indicating that variations in negative life events do not affect levels of wellbeing, or vice versa. Although three of the cross-lagged estimates reached statistical significance, the effects were weak. The highest estimate was observed for the cross-lagged path from negative dependent life events at Wave 1 to wellbeing at Wave 2, where fluctuations in negative dependent life events explained only 1% (i.e., $\:{-0.11}^{2}$) of the changes in wellbeing.

**Table 3:** Cross-lagged parameter estimates

	Standardized estimate
Life events → WB	
NegDep _Wave 1_ → WB _Wave 2_	**-0.11 (0.046)**
NegDep _Wave 2_ → WB _Wave 3_	-0.02 (0.045)
NegInd _Wave 1_ → WB _Wave 2_	-0.01 (0.041)
NegInd _Wave 2_ → WB _Wave 3_	-0.05 (0.040)
*WB → Life events*	
WB _Wave 1_ → NegDep _Wave 2_	**-0.08 (0.043)**
WB _Wave 2_ → NegDep _Wave 3_	**-0.10 (0.045)**
WB _Wave 1_ → NegInd _Wave 2_	-0.01 (0.038)
WB _Wave 2_ → NegInd _Wave 3_	-0.01 (0.038)

Standard errors in parentheses

*WB* wellbeing, *NegDep* negative dependent life events, *NegInd* negative independent life events

Significant path coefficients are presented in bold

## Discussion

The aim of this longitudinal twin study was to examine wellbeing during adolescence and young adulthood and to identify the relative influences of genetic and environmental factors in explaining stability and change. Using data from three measurement waves drawn from seven national cohorts (Wave 1: 12–18 years; Wave 2: 14–20 years; Wave 3: 16–22 years), we focused on within-individual, age-related change as observed in the sampled cohorts. We also explored the effects of life events as potential measured environmental influences on wellbeing within a genetic framework.

Cross-sectional heritability of wellbeing was approximately 50% at Waves 1–2 and declined to 26% at Wave 3. The remaining variance was attributed nonshared environmental factors, while the shared environment had no influence on the trait. Because the three waves include partially overlapping age ranges, the apparent decline in heritability should be interpreted cautiously and does not constitute a proof of a age-related decrease in heritability, even though participants were older at Wave 3. These results align with previous studies that have consistently documented moderate genetic effects on adolescent wellbeing, mainly accounting for trait stability (Bartels 2015; Baselmans et al. 2018; de Vries et al. 2024; Nes and Røysamb 2015). In line with this, the findings suggest that wellbeing demonstrates moderate stability within individuals across the sampled age windows, with stability largely attributable to genetic factors. Specifically, about 40% of the total variance in wellbeing at each wave was attributable time-invariant stability, of which over 80% was explained by genetic influences. These findings align with previous studies on wellbeing, and correspond with the genetic architecture found in most personality traits (Briley and Tucker-Drob 2014; McGue et al. 1993).

It is noteworthy that the genetic influences observed during adolescence in this sample were more pronounced compared to those reported in studies of earlier and later developmental stages. In childhood, shared environmental factors have shown some significant effects, while in adulthood, nonshared environmental factors become more prominent (Baselmans et al. 2018; de Vries et al. 2024). In the current study, genetic and nonshared environmental influences each accounted for roughly half of the variance in wellbeing during the first two measurement waves. However, by the final wave, when participants were 16 to 22 years, genetic influences had decreased significantly to explain around one-quarter of the variance, with the rest accounted for by nonshared environmental factors. This pattern has also been observed in other studies (Sisk and Gee 2022).

To interpret these results, it may be useful to examine what is already known about elements within the nonshared environment. In behavioral genetics, the nonshared environment is defined as the variance in a trait remaining after accounting for genetic and shared familial influences. Factors contributing to differences among individuals raised in the same family, also including measurement error, have traditionally been hypothesized to encompass distinctive friendships, individual life events, and personal relationships and experiences (Plomin and Dunn 2001). In the context of the present study, although partially overlapping age groups in the measurement waves prevent us from drawing definitive conclusions, our results suggest that levels of wellbeing tend to remain relatively stable over time. Unique environmental influences—potentially stemming from events related to education, career choices, social relationships, and personal experiences—may play an increasingly significant role in overall wellbeing as adolescents transition to adulthood (Lee and Yang 2022). Accordingly, we found that measures of negative dependent and independent life events consistently showed significant associations with wellbeing within each measurement wave. However, if life events indeed impacted wellbeing levels, we would anticipate significant within-time nonshared environmental correlations between these constructs using genetically informed random intercept cross-lagged analyses. The results showed that the observed concurrent nonshared environmental correlations were modest. Furthermore, the cross-lagged estimates from the RI-CLPM analyses of measured life events were also very small, indicating that changes in life events had minimal predictive power for wellbeing over time. Specifically, both cross-lagged effects from negative independent events on wellbeing were negligible and non-significant, whereas one cross-lagged effect from negative dependent events on wellbeing reached significance. However, this effect was modest: changes in the number of negative dependent events explained only about 1% of the variance in wellbeing at the subsequent measurement wave. Although very small, the effect is nevertheless non-zero.

This finding aligns with previous research demonstrating minimal and at best time-limited associations between different stressful life events and self-reported wellbeing (Howard et al. 2022). Our findings also align with studies on life stressors as components of nonshared environments, such as the recent study by Gidziela et al. (2023) reporting that environmental factors accounted for less than 2% of nonshared variance in predicting adult outcomes from childhood and adolescent behavior.

Importantly, the modest cross-lagged effects between our measured life events and wellbeing should not be taken to imply that nonshared environmental influences are unimportant or merely measurement error. An alternative and complementary interpretation is that nonshared environmental effects are highly person-specific: the same objectively reported event may have very different psychological consequences depending on individual susceptibility, context, timing, and appraisal. Such heterogeneity would reduce average, across-sample associations even if idiosyncratic environmental effects are consequential for particular individuals.

Despite the daunting complexity of the processes underlying genetic and nonshared environmental influences, understanding these mechanisms is essential for predicting persistent change and for enhancing mental health and wellbeing. Our results – showing strong genetic contributions to stability in wellbeing alongside increasing nonshared environmental influence across the sampled age windows—suggest that gene–environment interplay (including both gene–environment interaction and correlation) may develop and change over time. Such processes could amplify or attenuate genetic effects on wellbeing as individuals progress through adolescence and into young adulthood. Investigating developmental timing, and genetic mechanisms that drive these dynamics will be important for designing interventions that target modifiable influences on wellbeing.

To further enhance understanding of these mechanisms and to improve individualized prediction and intervention, research should build on behavior-genetic findings by combining idiographic approaches with advanced computational techniques and artificial intelligence (Levinson et al. 2025; Matthews et al. 2021). Complementary emphasis on biological processes, epigenetics, and subjective individual experiences (Plomin, 2024)may help identify targets for personalized prevention and treatment.

Because the measurement waves follow partly overlapping cohorts, the present findings describe within-individual age-related change rather than definitive cohort-independent transitions from adolescence into young adulthood. A limitation of the current design is that the three waves follow partly overlapping age cohorts (Wave 1: 12–18 years; Wave 2: 14–20 years; Wave 3: 16–22 years), with data drawn from seven national cohorts. Although participants age across waves, this structure means our primary findings pertain to within-individual change observed in these cohorts rather than cohort-independent developmental trajectories. Trajectories that follow younger adolescents may differ systematically from those that follow older participants, and our design does not directly test age-specific transitions into young adulthood. Future studies employing non-overlapping age cohorts, accelerated longitudinal designs that explicitly model cohort effects, or longer follow-up into later adulthood are needed to characterize developmental changes that are independent of cohort composition.

## Conclusion

Our findings document patterns of stability and change in wellbeing within individuals across the sampled adolescent and young-adult age. These findings emphasize the predominantly genetic basis of wellbeing stability during adolescence, while indicating that life events account for only a limited portion of the nonshared environmental variance. Nonshared environmental influences become more prominent as participants age across the sampled waves. These patterns should be interpreted as within-individual, age-related change rather than definitive, cohort-independent transitions into young adulthood.

## Supplementary Information

Below is the link to the electronic supplementary material.

Supplementary Material 1

## Data Availability

Data for the study was based on the Oslo University Adolescent and Young Adult Twin Project, a longitudinal project that commenced in 2005. The collection of health-related data was pre-approved in 2005 by the Norwegian Data Protection Authority (DPA) under a 20-year clause, later extended to 2028, of individual data protection and subsequent data deletion or anonymization. In light of these legal restrictions, data supporting this article could not be shared at the time of publication. Anonymized data may be requested after 2028.
