# Supplementary material for: Stability and Change in Wellbeing Throughout Adolescence and Its Relationship with Life Events: A Longitudinal Twin Study
Source: Behav Genet. 2026 Apr 11;56(2):99–109. doi: 10.1007/s10519-026-10262-4 (PMC13132952; doi:10.1007/s10519-026-10262-4)
Supplement: Supplementary file 1 — Supplementary Material 1 [file 10519_2026_10262_MOESM1_ESM.docx]

**Supplementary Materials**

**Online Resource**

**Title: Stability and Change in Wellbeing Throughout Adolescence and Its Relationship with Life Events: A Longitudinal Twin Study.**

**Table S1**

*Number of Twin Pairs*

|  | Complete pairs | |  | Incomplete pairs | |  |
| --- | --- | --- | --- | --- | --- | --- |
|  | MZ | DZ |  | MZ | DZ | Total twin pairs |
| Wave-1 | 492 | 797 |  | 15 | 40 | 1344 |
| Wave-2 | 346 | 558 |  | 30 | 82 | 1016 |
| Wave-3 | 241 | 368 |  | 78 | 163 | 850 |

*Note.* MZ = monozygotic twin pairs; DZ = dizygotic twin pairs.

**Table S2**

*The Life Events Scale*

| **Negative dependent life events** |
| --- |
| I had an important change in physical appearance, which upset me (acne, glasses, physical development, etc.) ^a^ |
| I was a victim of violence (mugging, sexual abuse, robbery) ^a^ |
| I was disappointed by a friend |
| I was disappointed by someone in the family |
| I did not get into a group or activity that I wanted to get into (music group, sports team, theater, etc.) ^a^ |
| I had major problems with a teacher |
| I did much worse than I expected in an important exam or course ^a^ |
| I had less contact with one of my parents ^a^ |
| I had many arguments with my siblings ^a^ |
| I had many arguments with my parents ^a^ |
| I was bullied by other pupils/adolescents |
| I broke up with a girlfriend/boyfriend ^a^ |
| I had an abortion (girls) / my girlfriend had an abortion (boys) |
| I lost a close friend ^a^ |
| **Negative independent life events** |
| I lost a pet |
| I changed schools ^a^ |
| I became seriously ill or was injured ^a^ |
| At least one parent or another family member became seriously ill or was injured ^a^ |
| One of my parents died ^a^ |
| A brother or sister died ^a^ |
| Another family member died ^a^ |
| One of my close friends died ^a^ |
| Mom or Dad’s friend moved in with us ^a^ |
| A member of my family ran away from home ^a^ |
| My parents divorced, moved apart ^a^ |
| One of my parents had problems at work ^a^ |
| One parent lost his or her job ^a^ |
| My mother began to work ^a^ |
| There has been a change in a parent’s job so that my parent is away from home more often ^a^ |
| The family financial situation was difficult ^a^ |
| There was some damage or loss of family property (such as apartment, house, car or bike) ^a^ |
| There were many arguments between the adults ^a^ |
| Someone in the family had problems with the police ^a^ |
| **Positive dependent life events** |
| I received a special award (trophy, diploma etc.) for something done at school ^a^ |
| I became more popular with my friends |
| I joined a fun group of friends |
| I got a boyfriend/girlfriend ^a^ |
| I got a new friend |

*Note.* ^a^ Question from the Life Event Questionnaire for Adolescents (LEQ-A; Masten et al., 1994). The wording in some of the questions were slightly changed from the LEQ-A.

**Figure S1**

*Genetically Informative Random Intercept Cross-Lagged Panel Model, With Descriptions of Calculations*


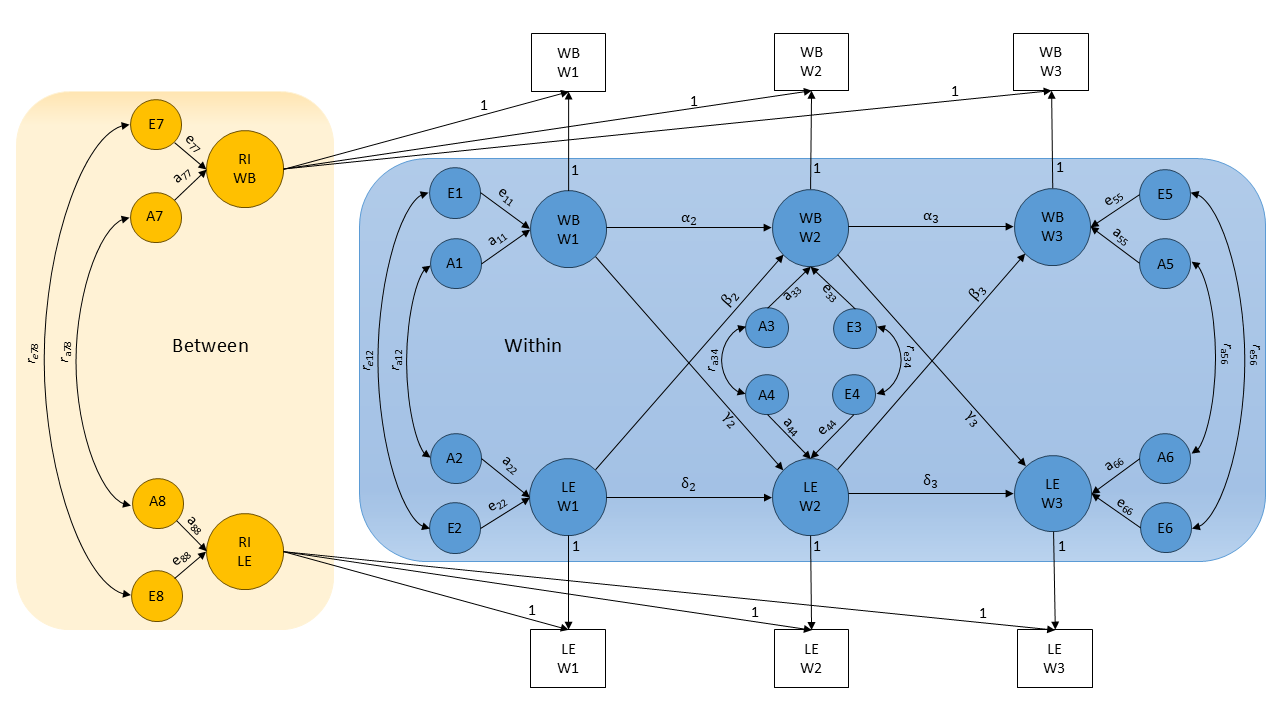


*Note.* The between‑person variance is the sum of genetic and environmental contributions to the random intercepts (wellbeing: $a_{77}^{2}+e_{77}^{2}$; life events: $a_{88}^{2}+e_{88}^{2}$). The within-person variance differs across waves; the example below shows the calculations for wellbeing (analogous calculations apply to life events). Within‑person variance in wellbeing at Wave 1 is simply $a_{11}^{2}+e_{11}^{2}$. At Waves 2 and 3, influences from both previous waves and new wave-specific influences contribute to the within-person variance. For example, the within‑person variance in wellbeing at Wave 2 equals the sum of:

- Stability effects (influences from previous wave): genetic: $\alpha_{2}^{2}+a_{11}^{2}$; environmental: $\alpha_{2}^{2}+e_{11}^{2}$
- Cross‑lagged effects (influence of prior life events on Wave-2 wellbeing): genetic: $\beta_{2}^{2}+a_{22}^{2}$; environmental: $\beta_{2}^{2}+e_{22}^{2}$
- Common/shared effects (joint Wave-1 influences on both traits): genetic: $2\times[\alpha_{2}\times a_{11}\times r_{a12}\times a_{22}\times\beta_{2}]$; environmental: $2\times[\alpha_{2}\times e_{11}\times r_{e12}\times e_{22}\times\beta_{2}]$
- Residual effects (new wave-specific influences): genetic: $a_{33}^{2}$; environmental: $e_{33}^{2}$​.

To calculate the proportion of variance explained by the random intercept, divide the between-person variance by the total variance (i.e., between-person variance + within-person variance). For Wave 1: ${Proportion}_{between, Wave 1}=\frac{a_{77}^{2}+e_{77}^{2}}{{(a}_{77}^{2}+e_{77}^{2})+{(a}_{11}^{2}+e_{11}^{2})}$

**Table S3**

*Descriptive Statistics*

|  |  | Wave-1 | | |  | Wave-2 | | |  | Wave-3 | | |
| --- | --- | --- | --- | --- | --- | --- | --- | --- | --- | --- | --- | --- |
|  |  | *n* | *M* | *SD* |  | *n* | *M* | *SD* |  | *n* | *M* | *SD* |
| Wellbeing |  | 2,588 | 7.74 | 1.51 |  | 1,892 | 7.50 | 1.52 |  | 1,442 | 7.44 | 1.57 |
| NegDep |  | 2,567 | 2.32 | 2.06 |  | 1,915 | 2.49 | 2.12 |  | 1,448 | 2.20 | 1.89 |
| NegInd |  | 2,627 | 1.44 | 1.36 |  | 1,918 | 1.46 | 1.43 |  | 1,453 | 1.39 | 1.37 |
| PosDep |  | 2,567 | 2.15 | 1.27 |  | 1,914 | 2.19 | 1.25 |  | 1,448 | 1.96 | 1.23 |

*Note.* NegDep = negative dependent life events; NegInd = negative independent life events; PosDep = positive dependent life events.

**Table S4**

*Heritability (a^2^) and Proportion of Unique Environmental Variance (e^2^) in Wellbeing After Regressing Each Wellbeing Measure On Within‑Wave Age*

|  | Model estimates | |
| --- | --- | --- |
| Variable | a^2^ | e^2^ |
| Wellbeing_1 | .47 [.41, .53] | .53 [.47, .59] |
| Wellbeing_2 | .46 [.39, .52] | .54 [.48, .61] |
| Wellbeing_3 | .26 [.16, .36] | .74 [.64, .84] |

*Note.* 95% confidence intervals in brackets. Numbers indicate the different measurement waves (1 to 3).

**Table S5**

*Model Fit Statistics of the Genetically Informative Random Intercept Cross-Lagged Panel Models*

| Model | CFI | TLI | RMSEA [95% CI] |
| --- | --- | --- | --- |
| Wellbeing and negative dependent life events | .981 | .982 | .014 [.007, .020] |
| Wellbeing and negative independent life events | .980 | .981 | .014 [.006, .019] |

*Note.* SWB = subjective well-being; CFI = comparative fit index; TLI = Tucker-Lewis index; RMSEA = root-mean-square error of approximation.

**Figure S2**

*Path Diagram of the Genetically Informative RI-CLPM With Unstandardized Coefficients for the Longitudinal Relationship Between Subjective Well-being and Negative Independent Life Events*


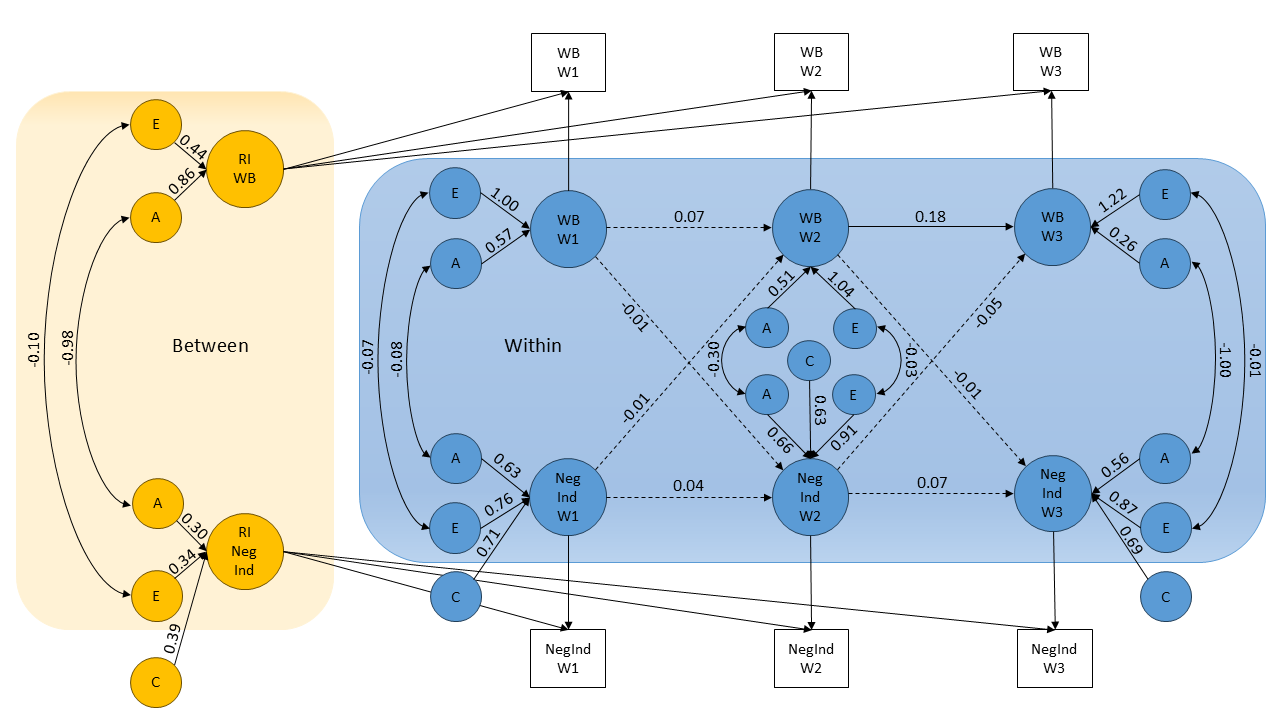


Note.  Dashed lines indicate nonsignificant paths. A detailed description of model parameters is provided in Figure 1. RI-CLPM = random intercept cross-lagged panel model; WB = wellbeing; NegInd = negative independent life events.

**Table S6**

*Unstandardized Parameter Estimates from the Genetically Informative Random Intercept Cross-Lagged Panel Models*

|  | | Wellbeing and NegDep | Wellbeing and NegInd |
| --- | --- | --- | --- |
| Autoregressive parameters | |  |  |
|  | WB_W1_ → WB_W2_ ($\alpha_{2}$) | 0.07 (0.06) | 0.07 (0.06) |
|  | WB_W2_ → WB_W3_ ($\alpha_{3}$) | 0.20 (0.05) | 0.18 (0.05) |
|  | LE_W1_ → LE_W2_ ($\delta_{2}$) | 0.23 (0.04) | 0.04 (0.05) |
|  | LE_W2_ → LE_W3_ ($\delta_{3}$) | 0.20 (0.04) | 0.07 (0.04) |
| Cross-lagged parameters | |  |  |
|  | WB_W1_ → LE_W2_ ($\gamma_{2}$) | -0.13 (0.06) | -0.01 (0.04) |
|  | WB_W2_ → LE_W3_ ($\gamma_{3}$) | -0.13 (0.06) | -0.01 (0.04) |
|  | LE_W1_ → WB_W2_ ($\beta_{2}$) | -0.07 (0.03) | -0.01 (0.04) |
|  | LE_W2_ → WB_W3_ ($\beta_{3}$) | -0.02 (0.03) | -0.05 (0.04) |
| Random intercept variance | |  |  |
|  | A WB (*a_77_*) | 0.86 (0.04) | 0.86 (0.04) |
|  | E WB (*e_77_*) | 0.41 (0.09) | 0.44 (0.08) |
|  | A LE (*a_88_*) | 1.14 (0.06) | 0.30 (0.16) |
|  | C LE (*c_88_*) | – | 0.39 (0.10) |
|  | E LE (*e_88_*) | 0.12 (0.11) | 0.34 (0.06) |
| Random intercept correlations | |  |  |
|  | A WB ↔ LE (*r_a78_*) | -0.46 (0.06) | -0.98 (0.52) |
|  | E WB ↔ LE (*r_e78_*) | -1.00 (1.08) | -0.10 (0.23) |
| Within-person variance | |  |  |
|  | A WB_W1_ (*a_11_*) | 0.59 (0.07) | 0.57 (0.08) |
|  | E WB_W1_ (*e_11_*) | 1.00 (0.05) | 1.00 (0.04) |
|  | A LE_W1_ (*a_22_*) | 1.04 (0.08) | 0.63 (0.11) |
|  | C LE_W1_ (*c_22_*) | – | 0.71 (0.08) |
|  | E LE_W1_ (*e_22_*) | 1.36 (0.04) | 0.76 (0.03) |
|  | A WB_W2_ (*a_33_*) | 0.49 (0.08) | 0.51 (0.08) |
|  | E WB_W2_ (*e_33_*) | 1.06 (0.04) | 1.04 (0.04) |
|  | A LE_W2_ (*a_44_*) | 0.88 (0.09) | 0.66 (0.14) |
|  | C LE_W2_ (*c_44_*) | – | 0.63 (0.12) |
|  | E LE_W2_ (*e_44_*) | 1.45 (0.05) | 0.91 (0.04) |
|  | A WB_W3_ (*a_55_*) | 0.26 (0.15) | 0.26 (0.17) |
|  | E WB_W3_ (*e_55_*) | 1.22 (0.04) | 1.22 (0.04) |
|  | A LE_W3_ (*a_66_*) | 0.65 (0.11) | 0.56 (0.19) |
|  | C LE_W3_ (*c_66_*) | – | 0.69 (0.12) |
|  | E LE_W3_ (*e_66_*) | 1.33 (0.05) | 0.87 (0.05) |
| Within-person correlations | |  |  |
|  | A WB_W1_ ↔ LE_W1_ (*r_a12_*) | -0.45 (0.12) | -0.08 (0.16) |
|  | E WB_W1_ ↔ LE_W1_ (*r_e12_*) | -0.19 (0.06) | -0.07 (0.06) |
|  | A WB_W2_ ↔ LE_W2_ (*r_a34_*) | -0.59 (0.18) | -0.30 (0.19) |
|  | E WB_W2_ ↔ LE_W2_ (*r_e34_*) | -0.12 (0.05) | -0.03 (0.06) |
|  | A WB_W3_ ↔ LE_W3_ (*r_a56_*) | -0.99 (0.65) | -1.00 (0.79) |
|  | E WB_W3_ ↔ LE_W3_ (*r_e56_*) | -0.10 (0.06) | -0.01 (0.06) |

*Note.* Standard errors in parentheses. LE refers to the respective life event cluster. WB = wellbeing; NegDep = negative dependent life events; NegInd = negative independent life events; W1, W2, W3 = wave-1, wave-2 and wave-3, respectively; A and a = additive genetic influences; C and c = shared environmental influences; E and e = non-shared environmental influences.
